# Lifestyle Effects on an Unusual Presentation of Syncope: A Case Report

**DOI:** 10.7759/cureus.69064

**Published:** 2024-09-10

**Authors:** Rishma Gattu, Nisarg Shah, Ramya Ravulapalli, Vania Rodriguez, Gary Merlino

**Affiliations:** 1 Osteopathic Medicine, Nova Southeastern University Dr. Kiran C. Patel College of Osteopathic Medicine, Davie, USA; 2 Internal Medicine, Mount Sinai Medical Center, Miami Beach, USA; 3 Internal Medicine, Nova Southeastern University Dr. Kiran C. Patel College of Osteopathic Medicine, Davie, USA

**Keywords:** bradycardia, cannabis, hypotension, marijuana, neurocardiogenic, parasympathetic, shift work, sleep, syncope, vasovagal

## Abstract

We present a 53-year-old Hispanic male with a history of palpitations and chronic marijuana use coming to the emergency department (ED) with three episodes of sudden loss of consciousness that occurred after starting his job as a night shift worker, which led to severe chronic sleep deprivation. These episodes lacked prodromal (chest pain, shortness of breath, palpitations, diaphoresis) and postictal (drowsiness, nausea, confusion, headache) symptoms. Electrocardiograms (EKGs) performed in the ED revealed sinus bradycardia with a heart rate of 54 beats per minute (bpm), which dropped to 37 bpm during admission. Overnight telemetry exhibited sinus pauses, characterized by a delay in atrial activity for at least three seconds. A repeat EKG showed an incomplete right bundle branch block (RBBB). The patient received a final diagnosis of recurrent syncope and was given an implantable loop recorder (ILR). The ILR revealed several sinus pauses over the span of three months with no syncopal episodes. The patient was educated on dietary and lifestyle modifications to reduce the risk of experiencing syncopal episodes. This case study explores a unique presentation of syncope with a multifactorial etiology and discusses the impact of lifestyle behaviors on syncope exacerbation.

## Introduction

Syncope, commonly known as fainting, is a transient loss of consciousness (LOC) due to a lack of perfusion to the brain. It is typically characterized by an abrupt onset, brief duration, and spontaneous recovery. Syncope is common in the general population, with a lifetime incidence greater than 35% [1]. To further illustrate the impact of syncope on the healthcare system, 30% to 40% of all patients presenting with syncope to the emergency department (ED) are usually admitted for further management, costing around 2.4 billion dollars in Medicare costs [1]. This condition is prefaced by presyncope, which includes nausea, dizziness, blurred vision, pallor, diaphoresis, and/or palpitations [2]. Syncope resolves through maneuvers that increase perfusion back to the brain, such as lying supine or receiving fluids. Causes of syncope are typically orthostatic, reflexive, or cardiac in origin [3].

Orthostatic hypotension is a drop of at least 20 mL of mercury (mmHg) in systolic blood pressure or at least 10 mmHg in diastolic blood pressure upon standing due to a maladaptive response from the vasculature and baroreceptors. Conditions that can cause orthostatic hypotension include hypovolemia due to diuresis or hemorrhage, adrenal insufficiency, and certain medications. A physical examination can rule out orthostatic hypotension by assessing blood pressure and heart rate changes after standing.

Reflexive syncope, also called vasovagal or neurocardiogenic syncope, results in a sudden decrease in heart rate and blood pressure, leading to diminished blood circulation to the brain and causing a temporary LOC. Usually, an inciting event, such as emotional distress, prolonged standing, and Valsalva maneuvers, can trigger this phenomenon. Situational syncope is a type of reflexive syncope in which defecating, urinating, eating, laughing, and coughing can cause fainting due to vagus nerve stimulation. A tilt table test helps to diagnose vasovagal syncope [3]. Another trigger of reflexive syncope includes carotid sinus hypersensitivity in older individuals, in which pressure on the carotid artery stimulates the baroreceptors to vasodilate the arteries and decrease blood flow to the brain suddenly. Carotid hypersensitivity can be ruled out via carotid sinus massage, where massaging fails to cause a syncopal episode.

Cardiac syncope can be related to arrhythmias or aortic stenosis. Conduction abnormalities within the heart can lead to sudden adaptive changes in vagal and sympathetic tones that can cause a drop in blood pressure or a change in heart rate. The function of the atrioventricular (AV) node is regulated by sympathovagal balance [4]. Sympathetic stimulation accelerates conduction and shortens the refractory period, while vagal stimulation exerts the opposite effect. Dual AV node physiology, consisting of slow and fast conduction pathways, predisposes individuals to conditions like AV nodal reentrant tachycardia (AVNRT) [4]. Although present in approximately half the population, dual AV nodes do not necessarily lead to AVNRT, as seen in our patient, who presented with bradycardia instead [5]. To rule out structural or conduction cardiac abnormalities, diagnostic tests include electrocardiography (EKG), an echocardiogram, a stress test, an electrophysiology study (EPS), and a Holter monitor.

## Case presentation

Patient information and presenting symptoms

A 53-year-old Hispanic male with a past medical history of palpitations and chronic marijuana use presents to the ED with complaints of three episodes of LOC. He denies any prodromal or postictal symptoms. He states he had two episodes of LOC one day prior to admission and one episode the night prior to admission. The patient states that he was sleeping and then woke up to go to the bathroom. He sat on the toilet to urinate and suddenly fainted with no prodromal symptoms. This occurred twice on the same day. He endorses a similar episode, which occurred the night before admission when he went to work for his night shift. At 1:30 AM, a witness stated that he saw the patient have LOC for two to three minutes. Upon waking, the patient returned to baseline immediately. He denied any chest pain, shortness of breath, headache, abdominal pain, incontinence, confusion, blurriness, or weakness. The patient reports his heart rate was 50 bpm at the time of the incident and that he slept 16 hours the prior day. The patient endorses feeling deprived of sleep since starting night shifts earlier in the year and has only been averaging four to six hours of sleep per night. He states he has had intermittent palpitations since he started working the job. The patient reports his palpitations cease spontaneously and are not associated with other symptoms. He also endorses a 15-pound weight loss in the past three months with no changes in diet. Upon entering the ED, the patient was asymptomatic. Vital signs were unremarkable, including orthostatics. The patient’s complete medical history includes depression, hemorrhoids, hepatitis, hiatal hernia, and pancreatitis. He also reported daily marijuana use; however, the quantity is unknown.

Diagnostics

**Figure 1:**
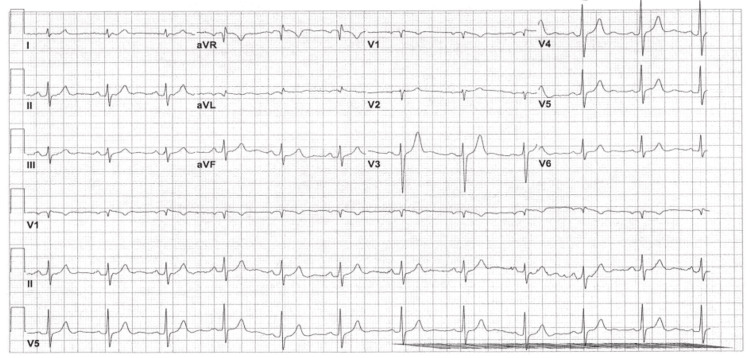
EKG done at 06:56 on day 1 of admission, showing borderline EKG with nonspecific intraventricular conduction block. EKG = electrocardiogram

To rule out common causes of syncope, as mentioned above, a complete metabolic panel was ordered in the ED, which was unremarkable, with no evidence of electrolyte abnormalities or hypoglycemia. To rule out cardiac etiologies, an EKG and troponins were ordered. Troponins were negative, ruling out an acute myocardial infarction. EKG showed a nonspecific intra-ventricular conduction block and a normal ventricular rate of 69 bpm (Figure 1).

**Figure 2:**
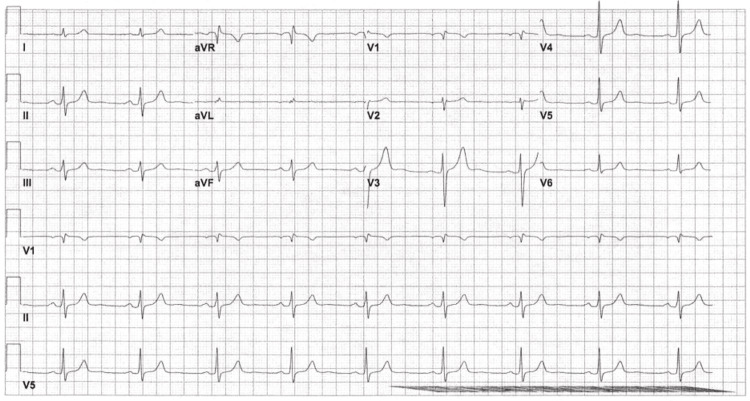
EKG done at 08:49 on day 1 of admission, showing borderline EKG with sinus bradycardia and incomplete RBBB. EKG = electrocardiogram; RBBB = right bundle branch block

EKG was repeated to confirm this finding, and the repeat EKG showed sinus bradycardia with a ventricular rate of 54 bpm with no change in the conduction block (Figure 2).

**Figure 3:**
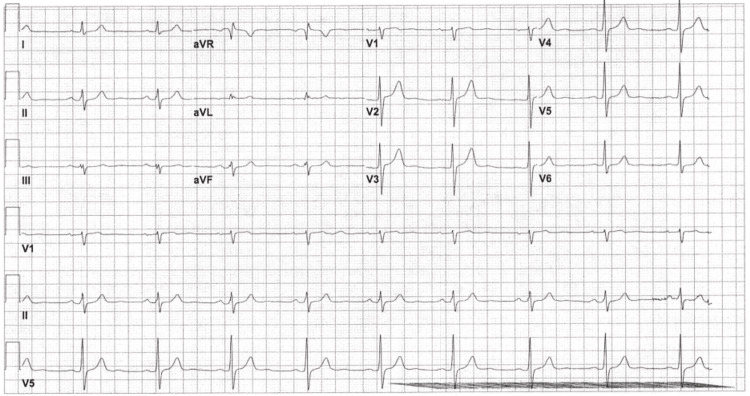
EKG done at 14:43 on day 1 of admission, showing normal EKG with sinus bradycardia and resolution of incomplete RBBB. EKG = electrocardiogram; RBBB = right bundle branch block

Another EKG was done two hours later to monitor changes, and it continued to show sinus bradycardia with a ventricular rate of 55 bpm, but it also showed the resolution of the conduction block (Figure 3).

**Figure 4:**
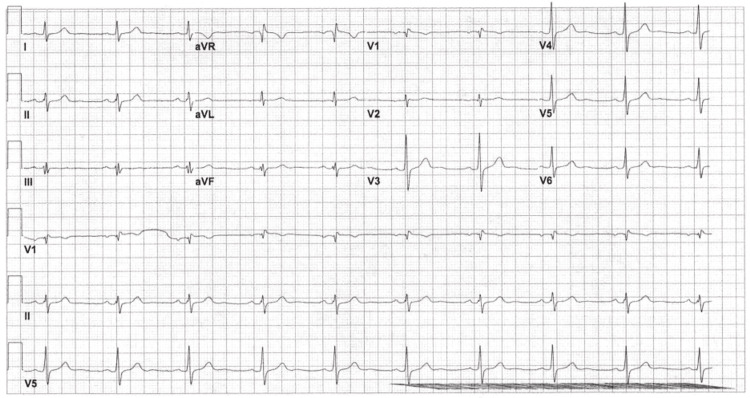
EKG done on day 2 of admission, showing borderline EKG with sinus bradycardia and incomplete RBBB. EKG = electrocardiogram; RBBB = right bundle branch block

A head computed tomography (CT) scan was also ordered to rule out any central neurological causes of the syncope, which was unremarkable. The patient was admitted for observation and further evaluation and placed on telemetry. Telemetry during the first night of admittance revealed an eight-second pause and a heart rate of 37 bpm at 2:00 AM, but the patient was reported to be asymptomatic. The patient endorsed poor sleep, as he had to go to the bathroom three times overnight to urinate, but he denies any episodes of LOC. The patient states he has been urinating more but has a weaker stream. He states he had a sensation of diffuse hyperspasticity, but his wife denies seeing any movement such as that overnight. On day 2 of admission, a repeat EKG was done, showing sinus bradycardia and incomplete right bundle branch block (RBBB) (Figure 4).

**Figure 5:**
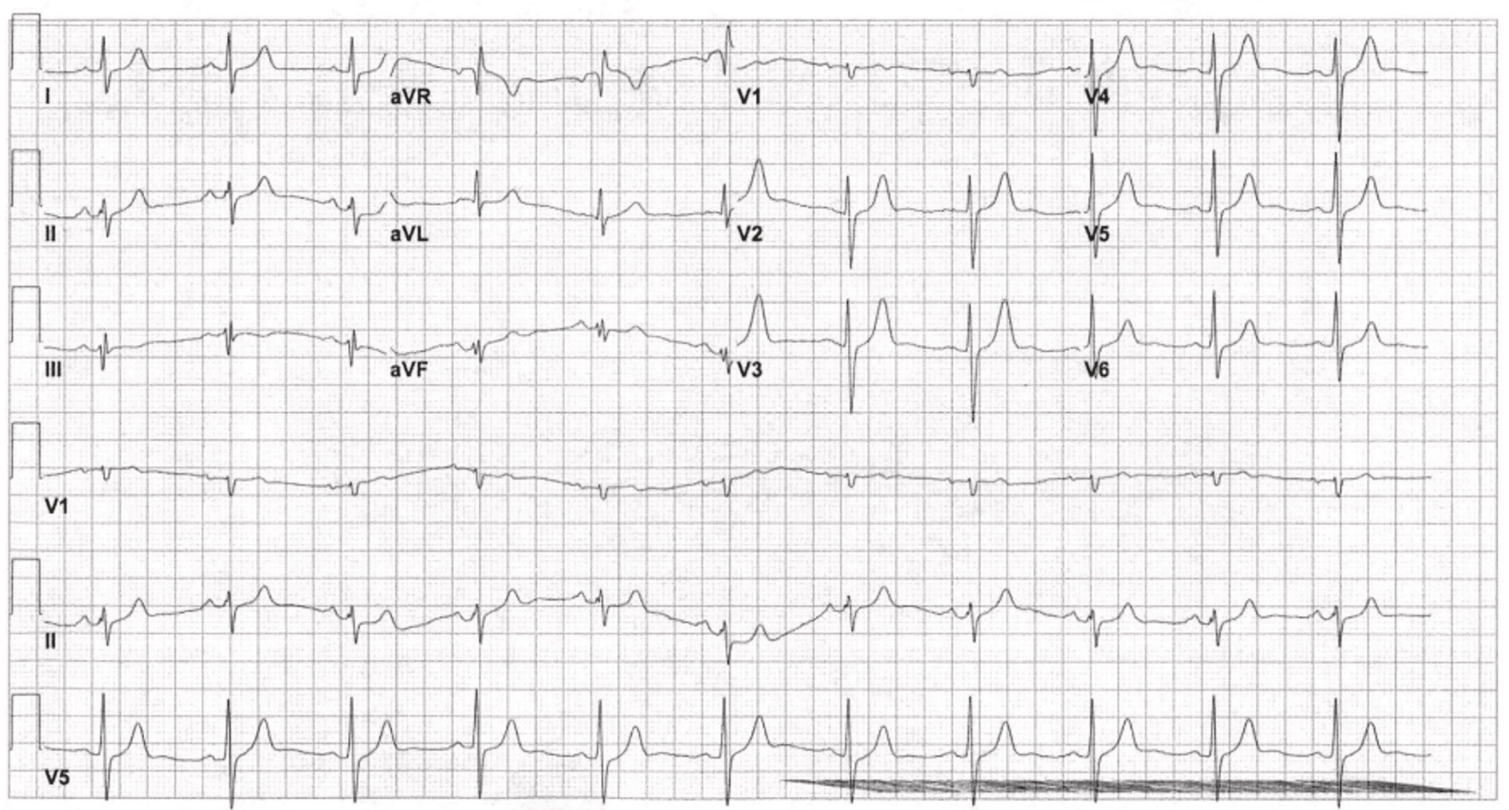
EKG done on day 3 of admission showing abnormal EKG, normal sinus rhythm, left axis deviation, and resolution of incomplete RBBB. EKG = electrocardiogram; RBBB = right bundle branch block

To further investigate cardiac etiologies, an EPS was conducted this day as well, which revealed a dual AV node (AVN) physiology but no accessory pathway. An echocardiogram was also conducted to identify any structural cardiac pathologies that may be causing the syncopal episodes; however, it was negative for any ventricular hypertrophy or valvular pathologies. An additional EKG was conducted on day 3 of admission to reassess any conductive pathologies, and it showed resolution of the conduction block that was present on the EKG during day 1 of admission (Figure 5).

Serial labs were also drawn to monitor the patient’s electrolyte status and hematological status (Table 1). 

**Table 1:** Lab results throughout the hospital course ALT = alanine aminotransferase; APTT = activated partial thromboplastin time; AST = aspartate aminotransferase; BUN = blood urea nitrogen; EGFR = estimated glomerular filtration rate; HCT = hematocrit; HGB = hemoglobin; INR = international normalized ratio; Mg = magnesium; PLT = platelets; TSH = thyroid-stimulating hormone; WBC = white blood cells

Lab	Reference	Day 1	Day 2	Day 3	Day 4	Day 5	Day 6	Day 7	Day 8
WBC	4.0-11.0 × 10³/µL	-	7.76	8.29	8.57	9.73	9.50	10.00	-
HGB	Males, 13.8-17.2 g/dL; females,12.1-15.1 g/dL	-	15.0	15.1	14.7	14.3	15.2	14.4	-
HCT	Males, 40.7%-50.3%; females, 36.1%-44.3%	-	45.2	44.7	43.3	42.2	45.6	42.4	-
PLT	150-450 × 10³/µL	-	284	278	270	269	280	277	-
Sodium	135-145 mEq/L	140	139	140	140	140	139	139	139
Potassium	3.5-5.0 mEq/L	4.4	4.3	4.4	4.0	4.0	4.4	4.3	4.5
Chloride	96-106 mEq/L	104	105	106	106	107	104	106	106
CO2	22-29 mEq/L	31.3	28	29	29	26	31	28	28
BUN	7-20 mg/dL	12	11	11	13	16	19	17	17
Creatinine/EGFR	0.6-1.3 mg/dL	0.88/>60	0.88/>60	0.86/>60	0.94/>60	0.90/>60	0.97/>60	0.86/>60	0.96/>60
Calcium	8.5-10.5 mg/dL	9.0	9.3	9.1	8.9	8.9	8.8	9.0	8.7
Glucose	70-99 mg/dL (fasting)	116	170	105	103	107	96	92	97
AST	10-40 IU/L	12	11	9	8	6	9	-	-
ALT	7-56 IU/L	16	14	14	14	14	13	-	-
INR	0.8-1.1	1.1	-	-	-	-	-	-	-
APTT	25-35 seconds	32.2	-	-	-	-	-	-	-
Mg	1.7-2.2 mg/dL	-	-	-	-	-	2.2	2.2	2.3
TSH	0.4-4.0 mIU/L	1.900	-	-	-	-	-	-	-
Troponins	Troponin I < 0.04 ng/mL; troponin T < 0.01 ng/mL	4.20 (4.30, 4.70)	-	-	-	-	-	-	-

Several differential diagnoses were discussed, and appropriate diagnostic measures were considered to rule out certain etiologies. Arrhythmias, as the primary cause of the LOC, were ruled out by serial EKGs, a Holter monitor, and a negative family history. Orthostatic hypotension was ruled out by the change of less than 10 mmHg in the blood pressure when the patient went from a supine to a standing position. Electroencephalography to rule out seizures was abnormal, with findings of bilateral cerebral dysfunction with increased activity in the frontal, central, and temporal derivations but no epileptiform patterns. Aortic stenosis was also considered but ruled out due to the absence of murmur and normal echocardiogram. A transthoracic echocardiogram was performed to rule out hypertrophic obstructive cardiomyopathy (HOCM); it revealed no valvular disease and a normal ejection fraction of 60-65%. Negative troponins, no ST changes, and no chest pain aided in ruling out a myocardial infarction. Narcolepsy was also considered; however, a cataplectic state constitutes being awake as opposed to this patient’s LOC episode. The patient also had a normal thyroid-stimulating hormone (TSH) level consistent with euthyroid state. The patient’s EPS findings of a dual AVN physiology were also explored further for a potential AVNRT. AVNRT was not inducible with isoproterenol. 

Management

Taking into consideration that the patient did not have any episodes of LOC during his hospital course, an implantable loop recorder (ILR) was implanted to monitor the patient’s cardiac rhythm while at home to potentially unveil the cause of this patient’s LOC. During a follow-up encounter conducted over the phone one month after discharge, it was discovered that the ILR recorded a six-second pause, at which time the patient reported feeling light-headed and dizzy, which prompted him to lie down. There were also episodes of bradycardia recorded the next day with a heart rate averaging 40 bpm; however, this occurred while the patient was sleeping. A few months later, the ILR noted two four-second pauses. The patient reported to be awake during these times due to his work shift. The patient reported dizziness during these episodes as well but denied LOC. Upon further investigation, a vagal etiology was suspected due to the progressive elongation of beats prior to the pause. The patient returned for a follow-up a few months later due to three episodes of pauses, with the longest pause lasting 6.1 seconds; the patient's wife reported that the patient was sleeping during this time. The patient had follow-up diagnostic work done, for which the EKG showed normal sinus rhythm with left axis deviation and a decreased T-wave amplitude in anterior leads. An echocardiogram revealed a normal left ventricular ejection fraction, and an EPS failed to show any electrical conduction abnormalities.

Outcome

This patient received a final diagnosis of recurrent syncope with unknown etiology. His condition was managed with the placement of ILR, as well as an extensive educational component on the adverse effects of chronic marijuana use and poor sleep hygiene, and avoiding AV blocking agents, such as beta-blockers, non-dihydropyridine calcium channel blockers, antiarrhythmics, and digoxin. The patient denied getting another episode of LOC after discharge from the hospital.

## Discussion

Syncope, characterized by a transient LOC due to inadequate cerebral perfusion, can present a diagnostic challenge. This case report highlights an unusual presentation of syncope, investigating the interplay between autonomic nervous control, lifestyle factors, and cannabis use.

Disordered sleep

Sleep has been implicated in many physical and cognitive functions, and its impact on syncope has been gaining more traction. An interesting finding to pay attention to is that this patient’s episodes occurred at rest, including sitting and lying down. A study conducted by Raj et al. explores a possible explanation for this observation [6]. Supine vasovagal syncope, or sleep syncope, represents a unique subtype of vasovagal syncope occurring nocturnally during sleep with the absence of typical triggers associated with vasovagal syncope, such as upright posture or emotional stressors. The pathophysiology of sleep syncope remains elusive, though it may involve the activation of the vasovagal reflex cascade triggered by vagal efferent traffic. Gastrointestinal symptoms preceding syncopal episodes suggest a vagal origin, with subsequent hypotension upon assuming an upright posture culminating in syncope [6]. The patient reported that he fainted while urinating after waking up to use the bathroom in the middle of the night. Although his symptoms were not gastrointestinal in nature, the increased vagal tone during the night, during micturition, and after standing suddenly may have, in combination, contributed to his syncopal spells.

Hu et al. offer evidence of the circadian pacemaker's influence on vasovagal responses to head-up tilt, resulting in increased susceptibility to presyncope overnight [7]. While individuals with typical sleep-wake patterns may sleep through this vulnerable period without encountering postural stressors, those who remain awake or wake up during the night time, such as shift workers, military personnel, emergency workers, airline pilots, truck drivers, parents of infants, and individuals with conditions like nocturia, insomnia, or other sleep disorders, may be particularly at risk. This heightened risk of syncope among these populations underscores the potential implications for both personal and public safety [7]. The onset of symptomatic presentation in our patient only after starting his night shift occupation suggests a compounded effect of sleep deprivation and disrupted circadian rhythms on syncope risk.

Marijuana effects

Guimarães et al. discuss the potential link between marijuana and cardiac dysrhythmias [8]. Despite coronary blood flow constituting only a small portion of systemic blood flow, the retention of cannabis compounds within the heart for extended periods may justify their cardiac effects, particularly on the sinus node. Marijuana consumption can manifest in various clinical symptoms, including bradycardia, sinus pauses, and orthostatic hypotension. Although uncommon, vasovagal syncope has been reported as a potential side effect of cannabis, resulting in sinus pauses due to extreme vagal tone and the compound's affinity for adenosine receptors [8]. Our patient, a chronic marijuana user who smoked daily and oftentimes shortly before work, would occasionally experience LOC an hour after smoking, suggesting a possible relationship between vasovagal syncope and marijuana use.

Grieve-Eglin et al. describe sinus arrests after acute ingestion of marijuana. After smoking 1 g, the patient experienced dizziness and tingling around the mouth, arm, and chest. Despite the lack of toxicology screening, the temporal correlation between marijuana inhalation and symptoms suggests a potential causal link. This case study advises monitoring individuals with presyncope or syncope who use high doses of marijuana [9].

Menahem et al. present a case of sinus arrest in a patient with a congenital heart defect after consuming significant amounts of marijuana and attributed the episode to increased vagal tone [10]. Similarly, Licciardi et al. investigate chronic cannabis use and hypervagotonia. The patient in this article was uniquely similar to ours in that they experienced syncope at rest and a lack of prodromal events [11]. Cannabidiol (CBD) in cannabis interacts with adenosine receptors, mimicking adenosine effects and potentially contributing to bradyarrhythmias and heightened vagal tone. Given the long half-life of THC, high levels of cannabinoids can accumulate with repeated use, prolonging their effects and impacting heart function [11]. 

After consuming cannabis, vasovagal syncope is a concern due to increased vagal tone, which can lead to sinus bradycardia and hypotension, resulting in syncope. In severe cases, heightened vagal tone may lead to sinus arrest. Brancheau et al. illustrate the potential for hypervagotonia induced by cannabis consumption to trigger asystole, highlighting the need for further investigation into this relationship [12].

Animal models have elucidated the mechanism by which cannabinoids modulate cardiac autonomic tone, with cannabinoid receptor 1 (CB1) agonists inducing sympathetic inhibition and enhancing vagal tone, leading to bradycardia and hypotension [13]. The dose-dependent effects of the compound vary based on cardiac regional neuronal distribution, with vagal influences prominent in the sinoatrial (SA) node and sympathetic neurons more concentrated in the ventricles. Lower doses typically result in sympathetic stimulation, while higher doses tend to induce parasympathetic stimulation [13].

Mathew et al. suggest that in healthy subjects, marijuana can induce severe postural dizziness and syncope, with drops in pulse rate, cerebral artery blood velocity, and blood pressure. While our patient's presentation was more consistent with vasovagal mechanisms, orthostatic changes can also cause syncope with marijuana use [14].

The patient's syncopal episodes manifested only after initiating his night shift occupation and subsided toward the end of the hospital stay as his sleep patterns improved, and he ceased marijuana usage. This temporal association strengthens the connection between disrupted sleep schedules and marijuana consumption in precipitating his syncope.

## Conclusions

Although there are few studies directly correlating sleep deprivation and syncope, the outcomes of our case highlight a potential compounded effect of sleep disruption and chronic marijuana use on syncope, possibly due to depression of cardiac autonomic function. This case adds to the limited body of literature on sleep and marijuana affecting syncope. It underscores the importance of considering lifestyle factors, such as occupational demands and substance use, in the evaluation and management of syncope. It also calls for further research into the combined effects of sleep disruption and cannabis on cardiac autonomics to better understand and mitigate these risks.

## References

[1] Saklani P, Krahn A, Klein G (2013). Syncope. Circulation.

[2] (2024). Vasovagal syncope. https://www.mayoclinic.org/diseases-conditions/vasovagal-syncope/symptoms-causes/syc-20350527.

[3] (2024). Syncope (fainting). https://www.hopkinsmedicine.org/health/conditions-and-diseases/syncope-fainting.

[4] Mani BC, Pavri BB (2014). Dual atrioventricular nodal pathways physiology: a review of relevant anatomy, electrophysiology, and electrocardiographic manifestations. Indian Pacing Electrophysiol J.

[5] Gonzalez MD, Banchs JE, Moukabary T, Rivera J (2019). 21 - Ablation of atrioventricular junctional tachycardias: atrioventricular nodal reentry, variants, and focal junctional tachycardia. Catheter Ablation of Cardiac Arrhythmias (Fourth Edition).

[6] Raj PL, Sheldon RS, Lorenzetti D, Jardine DL, Raj SR, Vandenberk B (2022). Sleep syncope-a systematic review. Front Cardiovasc Med.

[7] Hu K, Scheer FA, Laker M, Smales C, Shea SA (2011). Endogenous circadian rhythm in vasovagal response to head-up tilt. Circulation.

[8] Guimarães F, Camões J, Pereira M, Araujo R (2021). Cannabinoids: a cause of severe bradycardia. Cureus.

[9] Grieve-Eglin L, Haseeb S, Wamboldt R, Baranchuk A (2018). Symptomatic sinus arrest induced by acute marijuana use. J Thorac Dis.

[10] Menahem S (2013). Cardiac asystole following cannabis (marijuana) usage--additional mechanism for sudden death?. Forensic Sci Int.

[11] Licciardi M, Utzeri E, Marchetti MF, Nissardi V, Cecchetto G, Montisci M, Montisci R (2023). Syncope and cannabis: hypervagotonia from chronic abuse? A case report and literature review. BMC Cardiovasc Disord.

[12] Brancheau D, Blanco J, Gholkar G, Patel B, Machado C (2016). Cannabis induced asystole. J Electrocardiol.

[13] Kariyanna P, Wengrofsky P, Jayarangaiah A (2019). Marijuana and cardiac arrhythmias: a scoping study. Int J Clin Res Trials.

[14] Mathew RJ, Wilson WH, Davis R (2003). Postural syncope after marijuana: a transcranial Doppler study of the hemodynamics. Pharmacol Biochem Behav.

